# Synchronizing immunity with time: unlocking the potential of chrono-immunotherapy in cancer

**DOI:** 10.3389/fimmu.2026.1887336

**Published:** 2026-08-13

**Authors:** Liping Li, Chenliang Zhang, Ting Zhang

**Affiliations:** 1 Department of Pharmacy, Chengdu Fifth People’s Hospital, Chengdu University of Traditional Chinese Medicine, Chengdu, China; 2 Division of Abdominal Tumor, Department of Medical Oncology, Cancer Center, Laboratory of Abdominal Tumor Immunology and Microenvironment, State Key Laboratory of Biotherapy, West China Hospital, Sichuan University, Chengdu, China; 3 Department of Medical Cell Biology and Genetics, School of Basic Medical Sciences, Southwest Medical University, Luzhou, China

**Keywords:** chronotherapy, circadian rhythm, immunotherapy, tumor immune microenvironment, tumor immunity

## Abstract

Circadian rhythms, an endogenous timing system that enables organisms to adapt to Earth’s rotation, profoundly regulate fundamental physiological processes including cellular metabolism, hormone secretion, and immune surveillance. Recent advances have revealed that circadian disruption drives tumor initiation and progression, with its modulatory role in tumor immunity serving as a pivotal link between basic chronobiology and clinical oncology. Building upon an overview of circadian concepts and molecular regulatory mechanisms, this review synthesizes the multidimensional influence of circadian rhythms on tumor immunity, encompassing the temporal dynamics of immune cell proliferation, migration, and activation, remodeling of the tumor immune microenvironment, and the rhythmic regulation of immune checkpoint molecule expression. Furthermore, we examine ongoing efforts and remaining challenges in chronotherapy-based approaches to cancer immunotherapy. This review highlights that the intersection of circadian rhythms and cancer immunotherapy is transitioning from proof-of-concept studies to clinical implementation, ushering in a new paradigm that considers not only the timing of treatment but also the optimization of therapeutic timing.

## 1 Introduction

Life has evolved under the constant pressure of the Earth’s rotation, which imposes predictable 24-hour cycles of light and darkness. This evolutionary pressure has given rise to the circadian rhythm, a conserved adaptation mechanism that orchestrates physiological processes in alignment with the day-night cycle (1, 2). In mammals, circadian rhythms are essential for homeostasis, regulating diverse functions including cellular metabolism, hormone secretion and immune activity. Disruption of these rhythms is associated with metabolic disorders, accelerated aging, neurodegenerative diseases and an increased risk of malignancy (3–6).

A growing body of evidence indicates that circadian rhythm is a critical determinant of tumor progression and therapy resistance, largely through its influence on tumor immunity (7–9). The circadian clock governs the activity and function of immune effector cells, as well as the immunomodulatory properties of cancer cells, thereby shaping the evolutionary trajectory of tumors (10, 11). Conversely, the intrinsic rhythmicity of the human immune system provides a therapeutic window for timing immunotherapy, a concept that has fueled growing interest in circadian-based chronotherapy. Nevertheless, the precise molecular mechanisms through which circadian clocks regulate tumor immunity remain incompletely defined, and clinically actionable strategies are not yet established. In this review, we systematically synthesize current knowledge of circadian regulation of tumor immunity, discuss the underlying molecular circuitry, and highlight recent advances in chronotherapy for cancer.

## 2 Core molecular mechanisms of circadian rhythm

The mammalian circadian system is organized into central and peripheral clocks. The central pacemaker resides in the suprachiasmatic nucleus (SCN) of the hypothalamus, which entrains to environmental light–dark cycles and synchronizes rhythms throughout the body (12, 13). The SCN transmits timing signals via neuroendocrine cues—including glucocorticoids, which peak during the active phase, and melatonin, which predominates during rest—to peripheral oscillators in tissues such as the heart, liver and immune system (13). In addition to SCN-driven synchronization, peripheral clocks are modulated by non-photic zeitgebers, including feeding, physical activity and temperature (14, 15). Thus, systemic circadian homeostasis emerges from the integration of extrinsic environmental cues and intrinsic endocrine signals.

**Figure 1:**
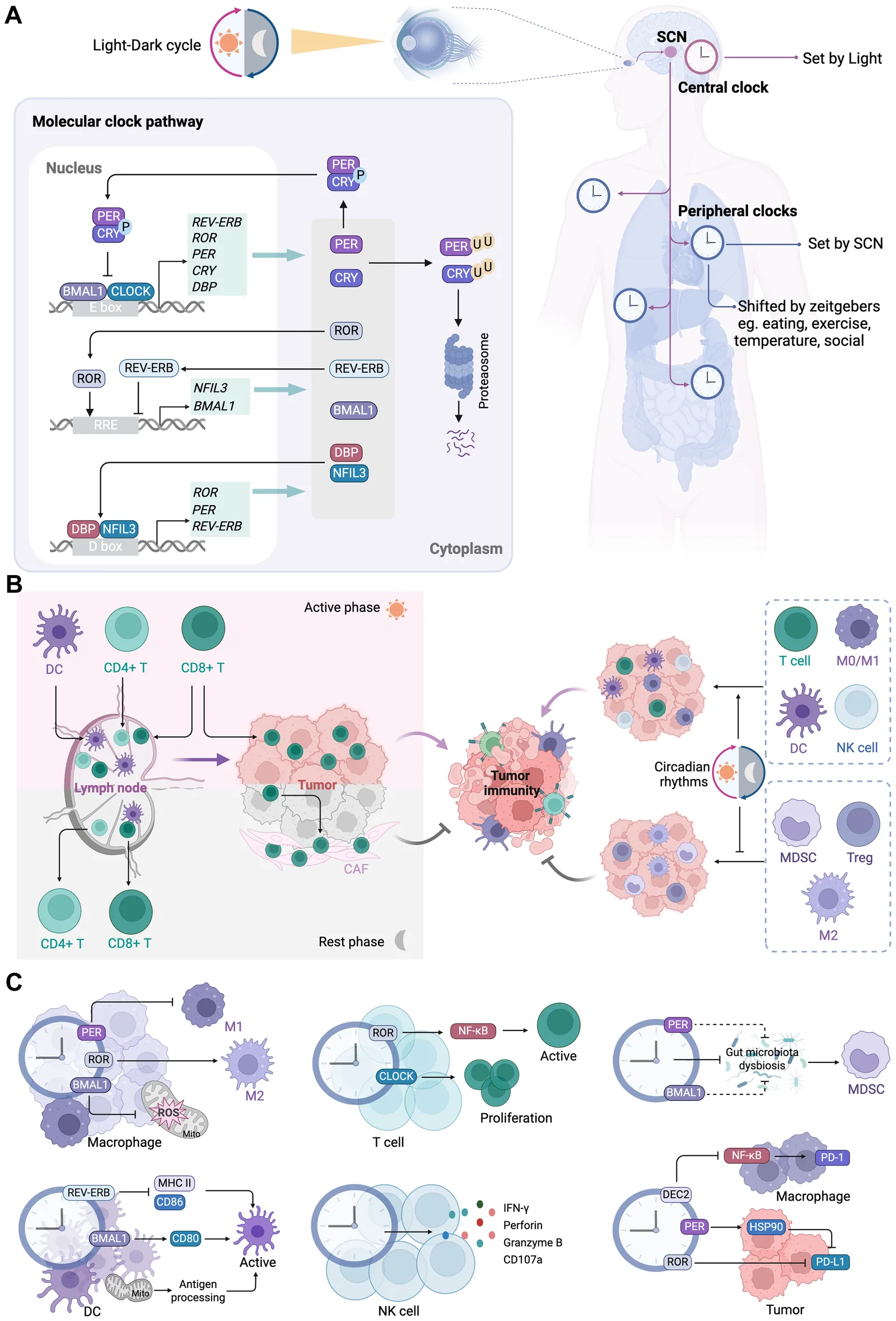
Circadian rhythm and its influence on tumor immunity **(A)** Circadian rhythm concept and core molecular regulatory mechanisms. Central Circadian Rhythm: Light signals are received by the eyes and transmitted to the suprachiasmatic nucleus (SCN). The SCN then regulates the central clock by modulating neuroendocrine signals. Peripheral Circadian Rhythm: The SCN transmits signals from the central clock to peripheral clocks via neuroendocrine signals, thereby regulating circadian rhythms in tissues such as the heart, liver, muscles, and intestines. Factors like eating, exercise, temperature, and social interaction can influence these peripheral rhythms. Molecular Clock Pathway: The BMAL1:CLOCK heterodimer promotes the transcription of clock-controlled genes, including *REV-ERB*, *ROR*, *PER*, *CRY*, and *DBP*, by binding to E-box elements. In the cytoplasm, accumulated PER and CRY proteins are phosphorylated by protein kinases, form heterodimers, and then translocate to the nucleus to inhibit the transcriptional activity of BMAL1:CLOCK. ROR and REV-ERB regulate the transcription of *BMAL1* and *NFIL3* by binding to RRE elements, acting as an activator and repressor, respectively. DBP and NFIL3 modulate the transcription of *ROR*, *PER*, and *REV-ERB* by interacting with D-box elements. **(B)** Circadian Regulation of Immune Cell Migration. During the active phase (daytime for humans), T cells and dendritic cells (DCs) migrate into lymph nodes and tumor tissue. During the rest phase (nighttime for humans), T cells and DCs exit lymph nodes; T cells within the tumor tissue migrate towards cancer-associated fibroblasts (CAFs). A normal circadian rhythm promotes the infiltration of pro-tumor immune cells, including T cells, M0/M1 macrophages, DCs, and NK cells, into the tumor tissue, while inhibiting the infiltration of immunosuppressive cells, including MDSCs, Tregs, and M2 macrophages. **(C)** Circadian Regulation of Immune Cell Function. In macrophages, the circadian regulator PER inhibits M1 polarization, ROR promotes M2 polarization, and BMAL1 inhibits mitochondrial reactive oxygen species (ROS) production, thereby maintaining macrophage activity. In T cells, ROR activates T cells by stimulating NF-κB, while CLOCK promotes T cell proliferation. In dendritic cells, REV-ERB inhibits DC activation by suppressing the expression of CD86 and MHC II. Conversely, BMAL1 promotes DC activation by enhancing CD80 expression and maintains mitochondrial fusion/fission dynamics to support efficient antigen processing, leading to DC activation. In NK cells, the circadian rhythm regulates the expression and secretion of cytokines and effector molecules, such as IFN-γ, Perforin, Granzyme B, and CD107a, thereby modulating NK cell activity. The circadian rhythm also influences the distal dissemination of tumor cells by regulating gut microbiota homeostasis and subsequently affecting the organ accumulation of MDSCs. The circadian regulator DEC2 suppresses PD-1 expression in macrophages by inhibiting NF-κB. In tumor cells, PER and ROR inhibit the expression of PD-L1. Created with BioRender.com.

At the molecular level, both central and peripheral clocks are driven by interlocked transcription–translation feedback loops. Key components include the transcriptional activators brain and muscle ARNT-Like 1 (BMAL1) and circadian locomotor output cycles kaput (CLOCK), their repressors cryptochromes (CRY1/2) and periods (PER1/2/3), and the nuclear receptors REV-ERBα/β (encoded by *NR1D1/2*), RORα/β/γ, and the D-Box binding PAR BZIP transcription factor (DBP). These core loop components are further regulated by post-translational modifications—including phosphorylation, dephosphorylation and ubiquitination—that control their stability, nuclear translocation and transcriptional activity (**Figure 1**) (16–19). The positive arm of the loop is initiated by BMAL1:CLOCK heterodimers, which bind to E-box elements (CACGTG) in the promoter regions of clock-controlled genes, thereby driving the expression of *CRY*, *PER*, *NR1D1/2* and *DBP* (19). Following translation, CRY and PER proteins accumulate in the cytoplasm, form heterodimers, and are phosphorylated by kinases, which promotes their nuclear translocation. Once in the nucleus, CRY: PER complexes compete with BMAL1:CLOCK for E-box occupancy, repressing their own transcription and completing the negative feedback loop (19, 20). Parallel regulatory loops add further precision. RORs bind to ROR response elements (ROREs) in the BMAL1 promoter and activate its transcription, an effect antagonized by REV-ERBs (21, 22). DBP interacts with D-box elements to regulate the expression of clock modulators, including *RORs*, *NR1D1/2* and *PER1/2*. DBP activity is opposed by NFIL3, which itself is transcriptionally controlled by RORs and REV-ERBs via ROREs in its promoter (19, 23). Together, these interlocked feedback loops ensure the robustness, amplitude and precise timing of circadian gene expression.

## 3 Circadian regulation of tumor immunity

Circadian disruption has emerged as a significant driver of tumorigenesis, a concern amplified by modern lifestyle factors such as artificial light at night, night-shift work, and irregular eating patterns. As these exposures become increasingly prevalent, the contribution of circadian dysregulation to cancer development will continue to grow, underscoring the importance of circadian-based strategies for cancer prevention and intervention. Accumulating evidence demonstrates that circadian disruption promotes tumor progression and malignant transformation through multiple mechanisms, including metabolic reprogramming, release of cell cycle restraints, maintenance of cancer stem cell properties, and facilitation of circulating tumor cell (CTC) generation to drive metastasis and recurrence (8, 9, 24). Importantly, beyond its direct effects on cancer cell-intrinsic properties, the circadian clock also shapes tumor evolution and therapeutic outcomes through its profound influence on anti-tumor immunity (**Figure 1**). Circadian oscillations in immune cell populations are a well-established biological phenomenon. In human peripheral blood, lymphocytes, monocytes and eosinophils show pronounced day–night differences in abundance and tissue trafficking (25, 26). The rhythmic entry of immune cells into the circulation, their egress, and their migration into tissues represent primary mechanisms through which the circadian system governs immune function. These oscillations inevitably extend to immune activity within the tumor microenvironment (TME), thereby modulating tumor progression (10). Indeed, both preclinical models and clinical specimens have revealed that circadian rhythms regulate the trafficking and functional states of diverse immune populations, including both pro- and anti-tumorigenic subsets, thereby shaping the TME and the overall anti-tumor immune response.

### 3.1 Circadian regulation of T cells

T cells are central effectors of anti-tumor immunity, capable of recognizing malignant cells and eliminating them through the release of cytokines and cytotoxic mediators such as perforin and granzymes (27). Circadian clocks influence multiple aspects of T cell biology, including proliferation, migration and differentiation. In mice, CD8+ T cells isolated from lymph nodes at different times of day exhibit distinct proliferative capacities upon stimulation, with cells collected during the night—the rodent active phase—showing the most robust expansion (28). This rhythmicity is abrogated in Clock-mutant mice, indicating that T cells possess an intrinsic circadian clock that regulates their proliferative potential. Druzd et al. demonstrated that in mice, CD8+ and CD4+ T cell numbers in the blood peak during the day (the rest phase) and reach a nadir at night, while lymph node T cells exhibit the opposite phase (29). Mechanistically, rhythmic expression of C-C motif chemokine receptor 7 (CCR7) on lymphocytes and its ligand CCL21 (C-C Motif chemokine ligand 21) in lymph nodes drives circadian T cell homing, whereas rhythmic expression of *sphingosine-1-phosphate receptor 1* (S1pr1) in lymph nodes governs T cell egress (29). This coordinated rhythmicity between lymphocytes and their microenvironment ensures proper temporal orchestration of T cell trafficking. Shimba and colleagues further showed that glucocorticoids regulate rhythmic expression of the interleukin-7 receptor (IL-7R) and the C-X-C motif chemokine receptor 4 (CXCR4) in T cells, thereby modulating their migration to lymph nodes (30). Extrapolating from nocturnal rodents to diurnal humans suggests that during the active phase (daytime in humans), T cells exhibit enhanced homing, proliferation and activation.

Circadian rhythms also impact T cell infiltration and function within tumors. In mouse tumor models, circadian disruption reduces CD8+ T cell infiltration while increasing the proportion of immunosuppressive myeloid cells in the TME—an effect associated with upregulation of the CXCL5–CXCR2 axis that results in enhanced tumor growth and metastasis (31). The BMAL1 transcriptional activator RORα potentiates CD8+ T cell anti-tumor activity by suppressing NF-κB signaling and enhancing cholesterol metabolism (32). Although it remains unclear whether this function of RORα is clock-dependent, these findings illustrate that circadian regulators can influence T cell activity through multiple mechanisms. Clinical correlative studies support these findings. In lung cancer patients with high risk scores for circadian disruption, infiltration of immune effector cells—including CD8+ T cells, dendritic cells (DCs), natural killer (NK) cells and M0/M1 macrophages—is significantly reduced, whereas immunosuppressive populations such as regulatory T cells (Tregs), M2 macrophages, mast cells and neutrophils are enriched (33). These observations suggest that circadian disruption may drive the TME toward an immunosuppressive state. Wang et al. demonstrated that in mice, CD8+ T cell infiltration into tumors is highest during the active phase and declines during the rest phase (34). Importantly, anti-PD-1 (programmed death receptor 1) and chimeric antigen receptor T-cell (CAR-T) therapy administered during the active phase exhibited superior anti-tumor efficacy, providing a rationale for chrono-optimization of immunotherapy (34). More recently, Tsuruta and colleagues uncovered an additional layer of complexity: circadian clocks regulate the migration of CD8+ T cells toward specific stromal subsets within tumors (35). They found that transforming growth factor-β (TGF-β)-SMAD signaling drives rhythmic expression of CXCR4 on CD8+ T cells, with peak expression at night, promoting their migration toward CXCL12-expressing cancer-associated fibroblasts (CAFs) and thereby dampening anti-tumor activity (35). Pharmacological inhibition of CXCR4 significantly enhanced the therapeutic efficacy of anti-PD-L1 (programmed death-ligand 1) (35). Taken together, these studies establish that T cell trafficking and function are under circadian control. Disrupting these rhythms impairs tumor immunity, whereas aligning therapeutic interventions with T cell circadian biology can enhance anti-tumor responses.

### 3.2 Circadian regulation of DCs

DCs are the principal professional antigen-presenting cells, responsible for sensing pathogen-associated and damage-associated molecular patterns (DAMPs) via pattern recognition receptors, upregulating major histocompatibility complex (MHC) class II (MHC-II) molecules, co-stimulatory molecules and pro-inflammatory cytokines, and thereby priming T cell responses (36, 37). In the context of cancer, DC activation promotes anti-tumor immunity and restricts tumor progression. DC migration is subject to circadian regulation. For example, the migration of DCs from the skin into lymphatic vessels exhibits diurnal rhythmicity, governed by BMAL1-dependent circadian expression of CCL21 in lymphatic endothelial cells (LECs), the adhesion molecule LYVE-1 on LECs, and the chemokine receptor CCR7 on DCs (38). Wang and colleagues further demonstrated that DC trafficking to tumor-draining lymph nodes follows a circadian pattern, resulting in rhythmic CD8+ T cell activation and time-of-day-dependent differences in tumor graft outgrowth (39). This effect is mediated in part by BMAL1-driven rhythmic expression of the co-stimulatory molecule CD80 on DCs, which engages CD28 on T cells to promote their activation (39). Immunotherapy administered in phase with this rhythm achieved superior efficacy. Circadian clocks also regulate DC activation state. Deletion of the BMAL1 transcriptional repressors REV-ERBα/β upregulates CD86 and MHC II expression on bone marrow-derived DCs, promoting their maturation (40). Conversely, pharmacological activation of REV-ERBα/β with SR9009 suppresses the expression of maturation markers and pro-inflammatory cytokines (40). Chloé C. Nobis and colleagues showed that during DC-driven CD8+ T cell activation, the circadian status of both DCs and T cells contributes to the efficiency of T cell priming (41). Moreover, BMAL1 in DCs regulates rhythmic mitochondrial fission and fusion, thereby influencing antigen processing (42). Given the central role of DCs in antigen presentation and T cell activation, circadian gating of DC function is a key determinant of T cell immunity. Optimal anti-tumor responses may require synchronization of circadian oscillators between DCs and T cells.

### 3.3 Circadian regulation of NK cells

NK cells are also subject to circadian control. Logan et al. reported that in rat models, circadian disruption alters the normal rhythmic expression of clock genes such as *Bmal1* and *Per* in NK cells, and concomitantly disrupts the rhythmic production of effector molecules including IFN-γ, perforin and granzyme B, thereby abrogating the circadian variation in NK cell cytolytic activity (43). Loss of this rhythmicity promotes lung cancer progression (43). Zeng et al. further showed in mice that circadian disruption accelerates NK cell senescence and reduces secretion of CD107a and IFN-γ, effects potentially mediated by suppression of the Eomes–CD122 axis—a key regulator of NK cell development and functional maturation (44). These alterations in NK cell biology translate into impaired immune surveillance: circadian disruption reduces NK cell-mediated clearance of MHC-I-deficient tumor cells and promotes melanoma lung metastasis (44).

### 3.4 Circadian regulation of macrophages

Macrophages are innate immune cells specialized in recognition, phagocytosis and antigen presentation, and they play complex, context-dependent roles in tumor immunity. Circadian clocks regulate multiple aspects of macrophage biology, most notably the rhythmic expression and secretion of Toll-like receptors, cytokines and chemokines, which confer diurnal variation in macrophage responsiveness (45–48). Circadian clocks also influence macrophage polarization. For instance, deletion of the circadian repressors Per1/2 promotes M1-like polarization, whereas activation of RORα favors M2-like polarization (48, 49). Given that pro-inflammatory M1 macrophages generally support anti-tumor immunity while anti-inflammatory M2 macrophages suppress it and promote tumor progression, circadian regulation of macrophage polarization has direct implications for cancer. Aiello et al. showed that chronic jet lag in mice accelerates tumor growth compared to normal light–dark housing conditions (50). Analysis of tumor-associated immune cells revealed no change in total macrophage numbers, but a significant reduction in M1-like macrophages and a decreased M1/M2 ratio in the jet-lagged group, correlating with enhanced tumor progression (50). BMAL1 in macrophages is a critical regulator of their anti-tumor function. In a co-injection tumor model, Bmal1 deletion in macrophages reduced CD8+ T cell infiltration and accelerated tumor growth—effects associated with impaired mitochondrial metabolism, specifically reduced oxygen consumption and increased reactive oxygen species (ROS) production (51). These findings suggest that mitochondria may serve as key effectors of circadian control over macrophage anti-tumor activity. Interestingly, Early et al. reported that Bmal1 in mouse macrophages directly binds E-box elements in the promoter of Nrf2—a transcription factor that suppresses immune responses by inhibiting ROS and repressing pro-inflammatory cytokine gene expression—thereby promoting *Nrf2* transcription (52). This suggests that circadian clocks may also exert anti-inflammatory effects in macrophages. Whether this contributes to tumor progression or therapy resistance remains to be determined. Tumor-associated macrophages (TAMs) are key drivers of the immunosuppressive TME. Knudsen-Clark et al. found that the acidic TME alters circadian gene expression in TAMs and disrupts their rhythmicity, promoting immune tolerance and tumor growth (53). Compared to wild-type TAMs, Bmal1-deficient TAMs exhibited enhanced pro-tumorigenic activity (53). Yoshida et al. demonstrated that noninvasive microcurrent stimulation could synchronize rhythmic clock gene expression in macrophages and enhance their phagocytic activity against tumor cells (54), suggesting that targeting macrophage circadian biology may have therapeutic potential.

### 3.5 Circadian regulation of MDSCs

Myeloid-derived suppressor cells (MDSCs) are a heterogeneous population of immunosuppressive cells, primarily of neutrophilic and monocytic origin, that arise from hematopoietic stem cells and potently suppress T, B and NK cell activity (55, 56). In cancer, MDSCs not only facilitate immune evasion but also promote angiogenesis, epithelial-mesenchymal transition (EMT), and metastasis (57–59). Circadian disruption promotes MDSC accumulation in tumors, reinforcing immunosuppression and accelerating tumor growth (60). Liu et al. recently uncovered a gut-brain axis-dependent mechanism linking circadian disruption to MDSC-mediated metastasis in colorectal cancer (CRC) (61). Chronic jet lag induced gut microbiota dysregulation, leading to MDSC accumulation in the lungs, reduced CD8+ T cell numbers, and enhanced CRC cell seeding. *Bmal1* or *Per1/2* deletion phenocopied the effects of chronic jet lag on pulmonary MDSC accumulation and CRC metastasis (61). In a melanoma lung metastasis model, rhythmic neutrophil infiltration into the pre-metastatic niche is a key determinant of metastatic burden (62), although the precise molecular mechanisms remain to be elucidated. Neutrophils can promote metastasis by releasing neutrophil extracellular traps (NETs)—extracellular scaffolds of chromatin DNA and proteins that capture CTCs and facilitate their extravasation (63). Given that circadian clocks also regulate CTC generation (64), it is plausible that circadian disruption promotes metastasis by simultaneously enhancing CTC shedding and neutrophil-mediated entrapment.

### 3.6 Circadian regulation of the tumor immune microenvironment

Tumor immunity unfolds within the complex ecosystem of the TME, involving interactions among immune cells, non-immune stromal cells, extracellular components and malignant cells (65, 66). As detailed above, circadian clocks govern the proliferation, functional state and infiltration of immune cells. However, circadian regulation extends to non-immune components of the TME, further shaping the immunological landscape. Tumor endothelial cells are central to angiogenesis and immune cell trafficking, and they play key roles in tumor progression, metastasis and TME remodeling (67). Adhesion molecules such as intercellular cell adhesion molecule-1 (ICAM-1) and vascular cell adhesion molecule 1 (VCAM-1) on endothelial cells exhibit circadian expression patterns, and their rhythmicity, together with rhythmic expression of pro-migratory factors (e.g., CD11α, CXCR4, CD49d) on leukocytes, coordinates the circadian rhythm of leukocyte homing (68). Qin et al. recently developed an endothelial cell circadian gene signature comprising 101 genes and showed that dysregulation of this signature correlates with poor prognosis in cancer patients (69). High scores were associated with enhanced immune infiltration, chemokine expression and therapeutic sensitivity (69), underscoring the importance of endothelial circadian rhythms in shaping the TME.

Circadian clocks in cancer cells themselves also influence the TME by regulating gene expression and the secretion of factors that modulate immune activity. For example, circadian clocks regulate rhythmic expression of angiogenic factors in tumor cells. In hypoxic tumor cells, vascular endothelial growth factor (VEGF) expression exhibits circadian variation, and the negative regulators PER2 and CRY1 suppress hypoxia-induced VEGF promoter activity in implanted tumors (70). In CRC cells, high CLOCK expression upregulates tumor angiogenesis-related genes including hypoxia-inducible factor 1-α (HIF-1α), aryl hydrocarbon receptor nuclear translocator (ARNT) and VEGF (71). In breast cancer cells, elevated CLOCK suppresses CD8+ T cell infiltration and promotes M2 macrophage polarization; mechanistically, CLOCK acetylates NF-κB, enhancing its transcriptional activation of PD-L1 (72). These effects collectively contribute to an immunosuppressive microenvironment. In glioblastoma (GBM), CLOCK maintains glioma stem cell (GSC) stemness and promotes microglial infiltration, thereby fostering an immunosuppressive TME (72). This pro-tumorigenic effect is mediated in part by CLOCK-driven transcriptional upregulation of olfactomedin like 3 (OLFML3), an olfactomedin family protein that recruits immunosuppressive microglia (73). Recently, studies reported that circadian rhythm influences the left-sided versus right-sided heterogeneity of CRC. Right-sided CRCs exhibited higher circadian disruption scores, which correlated with enhanced communication between cancer cells and pro-tumorigenic myofibroblastic CAFs (74). The authors identified Non-POU domain-containing octamer-binding protein (NONO) as a key regulator of this process, although the underlying molecular mechanisms remain to be fully elucidated. Another important route through which circadian rhythms shape the TME is via modulation of the gut microbiota. Circadian disruption induces gut microbiota dysbiosis, altering microbial diversity and metabolite profiles. These changes influence both innate and adaptive immune responses and remodel the TME (75, 76). For instance, pancreatic translocation of gut microbiota has been implicated in the formation of an immunosuppressive microenvironment (77). The relationship between circadian rhythms and the gut microbiota is bidirectional: circadian disruption suppresses beneficial bacteria and promotes harmful species, while microbial metabolites in turn influence circadian rhythms, particularly in peripheral tissues (78). Xu et al. provided a comprehensive review of the crosstalk between circadian rhythms and the TME, highlighting the importance of the gut microbiota as a mediator and discussing potential therapeutic strategies targeting this axis (76).

### 3.7 Circadian regulation of PD-1 and PD-L1 expression

PD-1, broadly expressed on T cells, NK cells, DCs and other immune cells, and its ligand PD-L1, primarily expressed on cancer cells and antigen-presenting cells, constitute a major immune checkpoint axis (79, 80). Engagement of PD-1 by PD-L1 suppresses T cell activity and promotes immune evasion, and immune checkpoint inhibitors (ICIs) targeting this pathway have revolutionized cancer immunotherapy (80). Circadian clocks regulate the expression of both PD-1 and PD-L1, representing an important mechanism through which circadian rhythms influence tumor immunity. In a mouse melanoma model, the circadian regulator Dec2 in TAMs suppresses NF-κB-driven transactivation of the *Pdcd1* gene (encoding PD-1), thereby modulating PD-1 expression levels and their rhythmicity (81). At ZT14 (the active phase for mice, corresponding to early morning in humans), Bmal1 and Dec2 expression reach a nadir, while Pdcd1 expression peaks (81). In oral squamous cell carcinoma cells, the circadian repressor PER2 suppresses PD-L1 expression and enhances T cell activity (82). Intriguingly, this function of PER2 is independent of its transcriptional regulatory activity. Instead, PER2 binds to HSP90, disrupting its interaction with inhibitors of kappa B kinase (IKKs) and thereby inhibiting NF-κB signaling and downstream PD-L1 expression. Overexpression of PER2 significantly enhanced the anti-tumor efficacy of the PD-L1 antibody durvalumab (82). Liu and colleagues further identified the positive circadian regulator RORα, which is downregulated in melanoma, as a key modulator of tumor immunity (83). Mechanistically, RORα binds the *PD-L1* promoter and suppresses its transcription, thereby enhancing anti-tumor T cell cytotoxicity. Pharmacological activation of RORα significantly potentiated the anti-tumor effect of CTLA-4 blockade in a mouse melanoma model (83). Taken together, these findings establish that PD-1 and PD-L1 expression is under circadian control, providing a mechanistic rationale for optimizing the timing of ICI administration to align with peak therapeutic vulnerability. Nevertheless, the regulation and rhythmic characteristics of PD-1 and PD-L1 expression differ across tumor types, posing new challenges for the application of chrono-optimized precision ICI therapy.

## 4 Clock-oriented immunotherapy

Although the impact of circadian rhythms on tumor progression and therapeutic response has been elucidated largely within the past decade, the concept of chronotherapy in oncology is far from new. Early applications aimed to optimize the timing of chemotherapy and radiotherapy. However, due to tumor heterogeneity and the disruptive effects of cytotoxic treatments on circadian rhythms themselves, whether chronotherapy truly enhances the efficacy of conventional modalities remains debated. The recent resurgence of interest in chronotherapy, positioning it at the forefront of cancer treatment innovation, stems from its compelling rationale in the context of immunotherapy, which is driven by the profound circadian regulation of immune cells and the tumor immune microenvironment (TIME).

### 4.1 Chronotherapy in ICI therapy

Given the widespread clinical adoption of ICIs, the potential of chronotherapy in this setting has been intensively explored (**Table 1**). In patients with non-small cell lung cancer (NSCLC), morning administration of nivolumab, an anti-PD-1 antibody, was associated with significantly longer progression-free survival (PFS) compared to afternoon infusion (84–86). Similar time-of-day effects on ICI efficacy have been observed in other malignancies, including advanced biliary tract cancer (87), gastric cancer (88), and melanoma (89). The prevailing interpretation is that appropriately timed ICI administration aligns with the rhythmic trafficking and activation of effector immune cells, such as CD8+ T cells, NK cells, and dendritic cells, which exhibit peak homing and functional competence during the active phase. Interestingly, Nomura et al. refined this concept by identifying the critical importance of the first ICI infusion. In recurrent or metastatic esophageal squamous cell carcinoma, patients whose initial dose was not administered in the morning failed to derive chronotherapeutic benefit, even if subsequent doses were morning-timed (90). This suggests that establishing synchronization between treatment and host immune rhythms from the outset may be essential for successful chronotherapy. However, not all ICI-treated tumors appear to benefit equally. In small cell lung cancer, for instance, no significant difference in overall survival or PFS was observed between morning and afternoon ICI administration (dichotomized at 15:00) (91), suggesting that tumor histology and immune microenvironmental composition dictate chrono-sensitivity.

**Table 1:** Clinical evidence for chrono-immunotherapy in cancer.

Drug(s)/therapeutic strategy	Cancer type	Intervention(s)	Outcome	Ref.
Nivolumab	Metastatic non-small cell lung cancer	Infusion timing: morning group (9:27–12:54) vs. afternoon group (12:55–17:14)	Morning group had significantly longer median PFS (11.3 months) and OS (34.2 months) than afternoon group (3.1 months and 9.6 months); morning group had 74% reduced risk of progression (HR = 0.26) and 83% reduced risk of death (HR = 0.17).	(84)
Tislelizumab, pembrolizumab, carrelizumab (monotherapy or combined with chemotherapy/anti-angiogenic agents)	Advanced non-small cell lung cancer	Infusion timing: morning group (before 12:00) vs. afternoon group (after 12:00)	Afternoon group had significantly longer PFS (16.5 vs. 9.8 months, HR = 1.87); benefit was more pronounced in the anti-angiogenic combination subgroup (HR = 3.60); no significant differences in ORR or DCR.	(85)
Immune checkpoint inhibitors	Advanced biliary tract cancer	Infusion timing: ≥20% of infusions after 16:30 vs. <20%	After 16:30 group had significantly shorter OS (matched: 10.1 vs. 14.5 months, HR = 1.80; pre-matched: 9.8 vs. 13.7 months, HR = 1.68) and shorter PFS (4.9 vs. 8.1 months, HR = 1.62); no significant differences in ORR or adverse events.	(87)
Immune checkpoint inhibitors (combined with chemotherapy)	Advanced gastric cancer	Infusion timing: ≥20% of infusions after 16:30 vs. <20%	Group with lower proportion of infusions after 16:30 had significantly longer OS (pre-matched: 21.4 vs. 15.4 months, HR = 1.64; matched: 22.4 vs. 15.4 months, HR = 1.82); no significant differences in PFS, ORR, or adverse events.	(88)
Atezolizumab, durvalumab (combined with chemotherapy)	Extensive-stage small cell lung cancer	Infusion timing: before 15:00 vs. after 15:00	Before 15:00 group had significantly longer PFS and OS; multivariable analysis identified early infusion as an independent prognostic factor (PFS: HR = 0.483; OS: HR = 0.373).	(91)
Radiotherapy, durvalumab	Locally advanced non-small cell lung cancer	Treatment timing: >50% of treatments within 3 hours of sunset vs. ≤50%	Radiotherapy during this window: 61% reduced risk of progression (HR = 0.39) and 73% reduced risk of distant metastasis (HR = 0.27); durvalumab infusion during this window: 113% increased risk of distant metastasis (HR = 2.13); chemotherapy timing showed no significant association.	(92)
Nivolumab, SBRT	Metastatic head and neck squamous cell carcinoma	Infusion/radiotherapy timing: classified as morning, midday, or afternoon	No significant differences in PFS, ORR, or grade ≥3 toxicity across different timing groups (PFS: morning 175 days vs. afternoon 58 days vs. midday 67 days, p=0.8).	(93)
CAR-T cells (predominantly CD19-targeted products)	Refractory B-cell malignancies	Infusion timing (per hour delay)	Each hour of delay was associated with a 24% increase in 90-day mortality, 17% increase in severe neurotoxicity, and 27% increase in mechanical ventilation; no significant association with severe CRS.	(98)
Ipilimumab + nivolumab	Advanced melanoma	Infusion timing: ≥50% of doses before 14:00 vs. after	Morning group: median PFS not reached vs. 7.8 months in afternoon group (HR = 0.29); morning group had significantly improved OS (HR = 0.25); afternoon group more frequently required systemic immunosuppressive therapy.	(99)
Immune checkpoint inhibitors (anti-PD-1/anti-CTLA-4)	Advanced cutaneous melanoma	Infusion timing: before 11:00 vs. after (≥50% of infusions)	No association between timing and efficacy (OS: HR = 0.94; PFS: HR = 0.99); however, severe adverse events were more common in the afternoon group (34% vs. 11%, p=0.004).	(100)
Pembrolizumab, nivolumab, atezolizumab, durvalumab, avelumab	Solid tumors (80% non-small cell lung cancer)	Infusion timing: morning (≤11:37) vs. afternoon	Morning infusion group had longer median OS (30.3 vs. 15.9 months); in PS0–1 patients, morning infusion was associated with higher response rate (58% vs. 41%) but also more frequent toxicity (49% vs. 34%); mortality risk ratio between worst timing (≈13:36) and early morning was 4.8.	(101)
Nivolumab, ipilimumab, pembrolizumab	Metastatic renal cell carcinoma	Infusion timing: ≥20% of infusions before noon (Group A) vs. <20% before noon (Group B)	Group A (higher proportion before noon) had significantly longer PFS and OS (PFS HR = 0.67; OS HR = 0.57); significant differences persisted after multivariable adjustment.	(102)

Adding further complexity, combination with radiotherapy may alter the rhythmic landscape of the TME. In one study, evening administration of the anti-PD-L1 antibody durvalumab following chemoradiotherapy yielded superior disease control (92). Conversely, in head and neck squamous cell carcinoma, no time-of-day dependency was observed for anti-PD-1 therapy combined with radiotherapy (93). These divergent findings suggest that radiotherapy itself may perturb immune circadian rhythms, complicating the extrapolation of chronotherapy principles from ICI monotherapy to combination regimens. A critical comparison of the available studies reveals substantial inconsistencies that challenge a one-size-fits-all chronotherapeutic approach. For ICI monotherapy, the time-of-day effect is robust in NSCLC (84, 85) and biliary tract cancer (87), but undetectable in small cell lung cancer (91), implying that tumor histology and baseline immune infiltrate composition are major determinants of chrono-sensitivity. Collectively, the evidence indicates that ICI therapy can benefit from chronotherapeutic optimization, but successful implementation requires detailed characterization of each patient’s immune rhythmicity. Future efforts should prioritize the development and validation of standardized methods for assessing patient immune circadian phase, enabling precise, personalized guidance of ICI timing to maximize clinical benefit.

### 4.2 Chronotherapy in vaccine therapy

Beyond ICIs, preliminary evidence suggests that chronotherapy may also enhance other emerging immunotherapeutic modalities, although studies remain limited. Tumor vaccines, which aim to introduce tumor-associated antigens and potentiate endogenous anti-tumor immunity, represent one such area (94). Preclinically, the timing of vaccination influences anti-tumor efficacy. In an ovalbumin (OVA)-based melanoma model, afternoon vaccination elicited stronger immune responses than morning administration (39). This effect was attributed to circadian regulation of dendritic cells, as Bmal1 deficiency in DCs ablated the time-of-day dependency of OVA vaccine-induced DC activation and CD8+ T cell infiltration (39, 42). Extrapolating from nocturnal rodents to diurnal humans, these findings suggest that morning vaccination in patients may be optimal. Indeed, clinical studies in non-cancer settings have demonstrated that daytime vaccination promotes more robust B cell activation and antibody production, supporting the translational relevance of circadian timing (95). Whether similar principles govern cancer vaccine efficacy, however, awaits dedicated clinical investigation.

### 4.3 Chronotherapy in adoptive cell therapy

Adoptive cell therapies (ACT), including CAR-T cells, have become cornerstone immunotherapies for hematologic malignancies and are expanding into solid tumors (96). Both preclinical and retrospective clinical analyses suggest that chronotherapy may enhance ACT efficacy (97). In a murine melanoma model, CAR-T cell infusion during the active phase (night for rodents) resulted in superior anti-tumor activity (34). Lyons et al. retrospectively analyzed CAR-T cell persistence and toxicity in patients as a function of infusion time, finding that earlier-day infusions were associated with greater efficacy, reduced severe neurotoxicity, and lower rates of mechanical ventilation compared to later infusions (98). These observations imply that the infiltration and function of adoptively transferred T cells remain subject to host circadian regulation. Collectively, while the data are preliminary, chronotherapy holds promise for optimizing cancer vaccines and ACT, and dedicated prospective trials are warranted.

### 4.4 Mitigating toxicity through chronotherapy

In addition to enhancing efficacy, reducing treatment-related toxicity is a critical goal, particularly for immunotherapies with narrow therapeutic windows. In clinical regimens involving infusion of the immune activator IFN-α, administration during the evening (20:00–22:00) did not compromise efficacy but significantly reduced adverse events, enabling higher tolerated doses (103). In mouse models, melatonin supplementation attenuated CAR-T cell-induced cytokine release syndrome and other toxicities without compromising anti-tumor effect (104). However, caution is warranted when extrapolating such findings to humans. Melatonin, as the hormonal messenger of darkness, aligns with the rest phase, which in diurnal humans corresponds to reduced T cell trafficking and activation—the very processes required for effective immunotherapy. Thus, while melatonin may dampen toxicity, its potential to blunt therapeutic efficacy must be rigorously evaluated. Harnessing chronotherapy to reduce toxicity therefore requires balancing the potentially opposing circadian optima for efficacy and safety.

### 4.5 Challenges and limitations for clinical implementation of chrono-immunotherapy

Despite encouraging evidence, translating chrono-immunotherapy into routine clinical practice faces substantial obstacles. A major challenge is inter-patient circadian variability, driven by genetic and lifestyle factors, which leads to marked differences in immune cell rhythms and undermines the value of generalized timing recommendations. Moreover, most current evidence derives from retrospective analyses, where confounding variables are prevalent, and many studies rely on coarse dichotomous cutoffs (e.g., morning vs. afternoon) that fail to capture individual endogenous phase. Prospective, phase-randomized trials are therefore urgently needed for independent validation. In addition, chronotherapeutic benefits vary across cancer types, further limiting broad applicability. Finally, circadian rhythms are susceptible to multiple perturbations, including patient behavioral patterns and conflicting chrono-optimization windows in combination regimens. Addressing these issues is essential for achieving meaningful clinical benefit from chrono-immunotherapy and expanding its therapeutic scope.

## 5 Conclusions and perspectives

Circadian rhythms, as an intrinsic temporal regulatory system, have emerged as critical determinants of immune surveillance and tumor progression. They govern the trafficking, activation, and proliferation of immune cells, remodel the TIME, and regulate the expression of immune checkpoint molecules. Chronotherapy, the strategic alignment of treatment timing with host circadian rhythms, has shown promising but still preliminary potential to enhance immunotherapy, as supported by early-phase retrospective studies, particularly for ICI outcomes. Nevertheless, the field confronts fundamental hurdles, including the need for validation through prospective randomized controlled trials, the predominance of retrospective evidence, the lack of standardized chronotyping protocols, inter-patient circadian variability, and uncertain applicability across different cancer types and combination regimens. Translating circadian mechanisms into personalized treatment strategies remains the pivotal challenge.

Future research should prioritize the following directions. First, the development of precise patient chronotyping and synchronization methodologies is essential. Much of our mechanistic understanding derives from nocturnal rodents, necessitating cautious extrapolation to diurnal humans. Deep characterization of human immune circadian regulation, coupled with portable, real-time monitoring technologies—such as wearable devices tracking temperature, activity, and hormonal fluctuations—will enable identification of individual “immunotherapeutic time windows” to guide treatment scheduling. Second, multifaceted circadian intervention strategies beyond simple treatment timing should be explored. Lifestyle interventions, including structured physical activity, environmental temperature modulation, time-restricted feeding, and photic manipulation, can enforce peripheral clock synchronization and optimize T cell homing, proliferation, and functional status. Pharmacologically, compounds targeting core clock components are advancing through preclinical and early clinical development, including CRY activators [e.g., KL001 (105), SH1705 (106)], REV-ERB agonists [e.g., SR9009 (107)], and BMAL1 modulators (108, 109). However, sustained activation or suppression of clock molecules may abolish rhythmicity itself. Achieving high-efficacy, low-toxicity intervention will require strategies that potentiate desired immune functions during specific phases while preserving overall circadian dynamics. Third, the challenge of “asynchrony” between immune cell and tumor cell circadian function must be addressed. Immune cells rely on rhythmicity for efficient migration and killing, whereas tumor cells may exploit circadian rhythms to fuel proliferation. Future precision interventions should aspire to cell-selective circadian modulation, for instance, using targeted delivery systems (nanocarriers, antibody–drug conjugates) to enrich clock-modulating agents in specific immune subsets (e.g., circulating T cells) while sparing tumor cells. This “immune-optimizing, tumor-ignoring” approach could achieve therapeutic gain without perturbing tumor clocks. Finally, the profound heterogeneity of tumor types and patient backgrounds must be confronted. Circadian gene expression profiles, immune infiltrates, and microenvironmental states vary markedly across malignancies, while genetic background, lifestyle, and social jet lag shape individual circadian phenotypes. This reinforces the importance of precise implementation of chronotherapy.

In summary, the convergence of circadian biology and tumor immunotherapy remains largely at the proof-of-concept stage, with early-phase retrospective and preclinical studies providing encouraging but not definitive evidence. By integrating individualized chronotyping, selective circadian modulation, and multi-dimensional intervention strategies, the current impasse of non-standardized chronotherapy may be gradually addressed, potentially improving the benefits of immunotherapy. Future prospective, phase-randomized trials and robust chronotyping tools are urgently needed to determine whether chrono-immunotherapy can deliver meaningful clinical benefit and to establish practical guidelines for its routine application.

## Glossary

SCN: suprachiasmatic nucleus
BMAL1: brain and muscle ARNT-Like 1
CLOCK: circadian locomotor output cycles kaput
CRY: cryptochrome
PER: periods
DBP: D-Box binding PAR BZIP transcription factor
ROREs: ROR response elements
CTC: circulating tumor cell
TME: tumor microenvironment
CCR7: C-C motif chemokine receptor 7
CCL21: C-C Motif chemokine ligand 21
CXCR4: C-X-C motif chemokine receptor 4
IL-7R: interleukin-7 receptor
NK: natural killer cells
Tregs: regulatory T cells
DCs: dendritic cells
PD-1: programmed death receptor 1
CAR-T: chimeric antigen receptor T-cell
TGF-β: transforming growth factor-β
PD-L1: programmed death-ligand 1
DAMPs: damage-associated molecular patterns
MHC-II: major histocompatibility complex class II
LECs: lymphatic endothelial cells
ICIs: immune checkpoint inhibitors
TAMs: Tumor-associated macrophages
MDSCs: Myeloid-derived suppressor cells
CRC: colorectal cancer
EMT: epithelial-mesenchymal transition
NETs: neutrophil extracellular traps
ICAM-1: intercellular cell adhesion molecule-1
VCAM-1: vascular cell adhesion molecule 1
VEGF: vascular endothelial growth factor
HIF-1α: hypoxia-inducible factor 1-α
GBM: glioblastoma
GSC: glioma stem cell
OLFML3: olfactomedin like 3
NONO: Non-POU domain-containing octamer-binding protein
NSCLC: non-small cell lung cancer
TIME: tumor immune microenvironment
ACT: Adoptive cell therapies
